# Inpatient Management of Bronchiolitis Before and After the COVID-19 Pandemic

**DOI:** 10.7759/cureus.96503

**Published:** 2025-11-10

**Authors:** Rachel Hammett, Yana Dimech, Sophie Grech, Julia Zahra, Chiara Grech, Patrick Sammut

**Affiliations:** 1 Department of Paediatrics, Mater Dei Hospital, Msida, MLT; 2 Department of Obstetrics and Gynaecology, Mater Dei Hospital, Msida, MLT

**Keywords:** acute bronchiolitis, covid 19, general paediatrics, inpatient care, respiratory infection

## Abstract

Introduction

Bronchiolitis is the most common cause of respiratory-related hospital admissions in children under two years. It is typically caused by viral infections, most commonly respiratory syncytial virus (RSV). Despite the availability of various international guidelines, Malta lacks local protocols, leading clinicians to rely on international recommendations for management.

Objectives

The primary aim of this audit was to compare epidemiological and management trends in bronchiolitis among children under one year admitted to Mater Dei Hospital between 2019 (pre-COVID) and 2024 (post-COVID). Secondary objectives included assessing adherence to the National Institute for Health and Care Excellence (NICE) guidelines on bronchiolitis management and evaluating the impact of the 2021 NICE guideline update.

Methodology

A retrospective audit was conducted using data from October to February of 2018-2019 and 2023-2024. Infants under 12 months diagnosed with bronchiolitis were included. Data was anonymised and extracted from medical records and the local portal for patient lab results. Statistical analysis was performed using Z-tests for proportions and Chi-squared test.

Results

A total of 196 patients were included - 90 in 2019 and 106 in 2024. Admissions increased post-COVID (p = 0.008), with a higher proportion of male patients in 2024. Typical bronchiolitis symptoms were more common in 2024, including coryza (p = 0.0214), spluttery cough (p < 0.001), and wheeze/crackles (p = 0.0085). Fluid intake <50% of usual amount became a more frequent reason for admission (p = 0.014).

Investigations increased significantly post-pandemic, with more chest X-rays, blood tests, and viral PCR screens performed (all p < 0.001), despite guideline recommendations to limit these. Non-evidence-based treatments were commonly used, with salbutamol prescribed in over 60% of cases in both years. There was also increased use of hypertonic saline (p = 0.0464), antibiotics (p < 0.001), and corticosteroids (prednisolone p < 0.001; hydrocortisone p = 0.0355) in 2024.

More patients required fluid support in 2024 (p = 0.0008), though intravenous fluids were favoured over nasogastric hydration, contrary to best practice. Oxygen requirement significantly increased post-COVID (p = 0.0001). Despite improvements in documentation, reflected by more admission plans and treatment charts including oxygen (p = 0.001 and p = 0.0084, respectively), 69% of oxygen-dependent patients in 2024 still did not have a formal oxygen prescription.

Conclusion

The audit identified suboptimal adherence to NICE guidelines in the management of bronchiolitis. Post-COVID, there was a notable increase in admissions, investigations, and non-guideline-supported treatments, suggesting a drift from evidence-based care. Improved documentation and the development of local protocols are essential to standardise care and reduce unnecessary interventions.

## Introduction

Bronchiolitis is the leading cause of lower respiratory tract infection and hospitalisation in children under two years of age worldwide. The disease burden is particularly high in infants younger than six months, who are more likely to develop severe disease requiring hospital care [1]. It is typically caused by viral infections. Respiratory syncytial virus (RSV) is the predominant pathogen, accounting for approximately 60-80% of cases, though other viruses such as human rhinovirus, parainfluenza, adenovirus, and metapneumovirus are also implicated [2]. Globally, bronchiolitis imposes a major public health burden, with RSV estimated to cause more than 30 million lower respiratory tract infections annually, resulting in over three million hospitalisations and approximately 60,000 deaths in children under five years, the vast majority occurring in low- and middle-income countries [3]. Seasonality is well-recognised, with peaks typically during the winter months in temperate climates [4]. Several evidence-based international guidelines are available, including those from the American Academy of Pediatrics (AAP), the National Institute for Health and Care Excellence (NICE, UK) and the Paediatric Research in Emergency Departments International Collaborative (PREDICT). These emphasise that management is largely supportive, with oxygen supplementation and hydration as the mainstay of care, with no role for routine use of bronchodilators, corticosteroids, or antibiotics [5-7]. In Malta, however, no national protocol currently exists, meaning clinicians rely on these international guidelines for diagnosis and management decisions. This lack of locally adapted guidance highlights a gap in standardised care.

## Materials and methods

Data were collected retrospectively over two five-month periods, spanning October 2018 to February 2019 and the same months in 2023-2024. The study population consisted of infants younger than one year who were admitted to Mater Dei Hospital with bronchiolitis. Children with viral-induced wheeze, or with overlapping features of viral-induced wheeze and bronchiolitis, were excluded.

Clinical information was extracted from medical notes and from the hospital’s electronic portal for laboratory results. The data gathered included patient demographics, presenting symptoms, admission criteria, recognised risk factors for severe bronchiolitis, and details of prescribed medications and investigations. Information on fluid requirements and the method of administration was also recorded, as well as oxygen requirements, including whether oxygen prescriptions were documented in the treatment chart and on the admission plan.

All patient data were anonymised before analysis. Statistical comparisons were carried out using Z-tests for proportions and Chi-squared tests.

## Results

Patient demographics

A comparison of patient demographics between 2019 and 2024 revealed a notable increase in the proportion of male patients admitted with bronchiolitis (53.3%; n=48 vs 71.7%; n=76), with a corresponding decrease in female admissions. The average age of patients increased slightly from 4.75 months (interquartile [IQR] 3) in 2019 to 5.6 months (IQR 6.75) in 2024, though this difference was not statistically significant (p=0.315) (Table 1).

**Table 1:** Sex distribution of patients in 2019 and 2024 Values are presented as number and percentage of total participants for each year (2019 n=90, 2024 n=106)

	2019 (n=90)	2024 (n=106)
Male	48 (53.3%)	76 (71.7%)
Female	42 (46.7%)	30 (28.3%)

Symptoms

In terms of clinical presentation, children admitted in 2024 were more likely to present with coryzal symptoms (90.6%; n=96 vs 78.9%; n=71, p=0.0214), spluttery cough (98.1%; n=104 vs 76.7%; n=69, p<0.001), and crackles/wheeze on auscultation (79.2%; n=84 vs 62.2%; n=56, p=0.0085). The presence of tachypnea was also more common in 2024 (70.8%; n=75 vs 57.8%; n=52), though this did not reach statistical significance (p=0.0576) (Table 2).

**Table 2:** Symptoms of patients in 2019 vs 2024 Values are presented as number and percentage of patients for each year (2019 n=90, 2024 n=106). P values compare the proportion of patients with each symptom between the two years.

Symptoms	2019 (n=90)	2024 (n=106)	P value
Coryzal symptoms	71 (78.9%)	96 (90.6%)	0.0214
Spluttery cough	69 (76.7%)	104 (98.1%)	<0.001
Tachypnea	52 (57.8%)	75 (70.8%)	0.0576
Crackles/wheeze on auscultation	56 (62.2%)	84 (79.2%)	0.0085

Criteria for admission

When examining admission criteria, there was an increase in the proportion of children admitted due to reduced fluid intake (<50% of normal), rising from 20.0% (n=18) in 2019 to 35.8% (n=38) in 2024 (p=0.014). Other criteria, such as apnea, hypoxia (SpO₂ <92%), and respiratory distress, did not significantly differ between the two years (Table 3).

**Table 3:** Criteria for hospital admission of patients with bronchiolitis in 2019 vs 2024 Values are presented as number and percentage of participants for each year (2019 n=90, 2024 n=106). P values compare the proportion of patients meeting each admission criterion between the two years.

Criteria for admission	2019 (n=90)	2024 (n=106)	P value
Apnea	5 (5.6%)	9 (8.5%)	0.427
SpO2 persistently <92%	15 (16.7%)	24 (22.6%)	0.296
Respiratory distress, RR > 70	53 (58.9%)	72 (67.9%)	0.189
Fluid intake <50% usual amount	18 (20.0%)	38 (35.8%)	0.014
Low threshold for admission due to risk factors	21 (23.3%)	30 (28.3%)	0.429
Criteria not followed	12 (13.3%)	8 (7.5%)	0.182

Risk factors

Risk factors for severe bronchiolitis were generally similar across both cohorts. However, parental concern or issues with carer competence were recorded in 4.5% (n=4) of cases in 2019 but not at all (n=0) in 2024 (p=0.028). No patients were admitted for immunodeficiency in either year (Table 4).

**Table 4:** Risk factors for severe bronchiolitis in 2019 vs 2024 Values are presented as number and percentage of patients for each year (2019 n=90, 2024 n=106). P values compare the proportion of patients with each risk factor between the two years.

Risk factors	2019 (n=90)	2024 (n=106)	P value
Chronic lung disease	3 (3.4%)	1 (0.9%)	0.237
Congenital heart disease	1 (1.1%)	2 (1.8%)	0.658
< three months in age	24 (27.3%)	26 (23.9%)	0.732
Prematurity <32 weeks	3 (3.4%)	7 (6.4%)	0.300
Neuromuscular problems	0 (0.0%)	1 (0.9%)	0.357
Social issues	1 (1.1%)	2 (1.8%)	0.658
Carer competence/parental concern	4 (4.5%)	0 (0.0%)	0.028
No risk factors	52 (59.1%)	70 (64.2%)	0.234

Treatment prescribed

Treatment practices showed a marked shift between 2019 and 2024. The use of hypertonic saline increased (65.6%; n=59 vs 78.3%; n=83, p=0.0464), while there was also a statistically significant rise in the prescription of antibiotics (3.3%; n=3 vs 34.0%; n=36, p<0.001), prednisolone (12.2%; n=11 vs 41.5%; n=44, p<0.001), and hydrocortisone (2.2%; n=2 vs 9.4%; n=10, p=0.0355). Use of salbutamol was more common in 2024 (63.2%; n=47 vs 52.2%; n=67), but the difference was not statistically significant. None of the patients were prescribed ipratropium bromide or montelukast in 2019 or 2024 (Table 5).

**Table 5:** Treatment prescribed to patients admitted with bronchiolitis in 2019 vs 2024 Values are presented as number and percentage of patients for each year (2019 n=90, 2024 n=106). P values compare the proportion of patients receiving each treatment between the two years.

Treatment prescribed	2019 (n=90)	2024 (n=106)	P value
Hypertonic saline	59 (65.6%)	83 (78.3%)	0.0464
Salbutamol	47 (52.2%)	67 (63.2%)	0.119
Antibiotics	3 (3.3%)	36 (34.0%)	<0.001
Adrenaline nebs	0 (0.0%)	1 (0.9%)	0.356
Prednisolone	11 (12.2%)	44 (41.5%)	<0.001
Hydrocortisone	2 (2.2%)	10 (9.4%)	0.0355

Investigations performed

Investigations were more frequently undertaken in 2024, with significant increases in chest X-rays (26.7%; n=24 vs 76.4%; n=81, p<0.001), blood tests (37.8%; n=34 vs 72.6%; n=77, p<0.001), and respiratory viral screens (82.2%; n=74 vs 99.1%; n=105, p<0.001) (Table 6).

**Table 6:** Investigations performed in patients admitted with bronchiolitis in 2019 vs 2024 Values are presented as number and percentage of patients for each year (2019 n=90, 2024 n=106). P values compare the proportion of patients who underwent each investigation between the two years. CXR: chest X-ray

Investigations	2019	2024	P value
CXR	24 (26.7%)	81 (76.4%)	<0.001
Blood tests	34 (37.8%)	77 (72.6%)	<0.001
Respiratory screen	74 (82.2%)	105 (99.1%)	<0.001

Fluid replacement

Supportive care also differed between the two time points. The need for fluid replacement was greater in 2024 (37.7%; n=40 vs 21.6%; n=19, p=0.0008), although the route of administration remained predominantly intravenous in both cohorts. Out of the 19 patients that required fluids in 2019, 18 (95%) received intravenous fluids and one (5%) received feeds via a nasogastric tube (NGT). Similarly in 2024, 37 out of 40 (92.5%) of the patients that received fluids had intravenous fluids whilst the remaining three (7.5%) patients received feeds via NGT (Table 7).

**Table 7:** Fluid replacement administered to patients admitted with bronchiolitis in 2019 vs 2024 Values are presented as number and percentage of patients for each year (2019 n=88, 2024 n=106). P values compare the proportion of patients who received fluid replacement between the two years.

Fluid replacement	2019 (n=88)	2024 (n=106)	P Value
Yes	19 (21.6%)	40 (37.7%)	0.0008
No	69 (78.4%)	65 (61.3%)	

Oxygen requirements

Oxygen requirements also increased significantly in 2024, with 30.5% (n=32) of patients requiring oxygen for SpO₂ <92% compared to 11.8% (n=10) in 2019 (p=0.0001) (Table 8).

**Table 8:** Oxygen requirements among patients admitted with bronchiolitis in 2019 vs 2024 Values are presented as number and percentage of patients for each year (2019 n=85, 2024 n=105). P values compare the proportion of patients with each oxygen requirement between the two years.

Oxygen requirements	2019 (n=85)	2024 (n=105)	P value
SpO2 <92%	10 (11.8%)	32 (30.5%)	0.0001
SpO2 >92%	75 (88.2%)	73 (69.5%)	

Oxygen prescribed on treatment chart

There was a statistically significant (p=0.0084) improvement in oxygen prescription on treatment charts between the two years. In 2019, none (n=0) of the patients had oxygen prescribed on their treatment chart, whereas this was recorded in 31% (n=9) of relevant cases in 2024. Despite this progress, the majority (69%; n=20) of patients in 2024 did not have oxygen prescribed on the treatment chart (Table 9).

**Table 9:** Oxygen prescription on the treatment chart of patients admitted with bronchiolitis in 2019 vs 2024 Values are presented as number and percentage of patients for each year (2019 n=10, 2024 n=29). P values compare the proportion of patients with prescriptions documented on the treatment chart between the two years.

Prescribed on treatment chart	2019 (n=10)	2024 (n=29)	P value
Yes	0 (0.0%)	9 (31.0%)	0.0084
No	10 (100.0%)	20 (69.0%)	

Oxygen written on admission plan

In both years, the majority of patients had oxygen documented on the admission plan, increasing from 70% (n=7) in 2019 to 96.6% (n=28) in 2024, representing a statistically significant improvement (p=0.001) (Table 10).

**Table 10:** Oxygen written on admission plan in patients admitted with bronchiolitis in 2019 vs 2024 Values are presented as number and percentage of patients for each year (2019 n=10, 2024 n=29). P values compare the proportion of patients with oxygen documented on the admission plan between the two years.

Oxygen written on admission plan	2019 (n=10)	2024 (n=29)	P value
Yes	7 (70.0%)	28 (96.6%)	0.0010
No	3 (30.0%)	1 (3.4%)	

## Discussion

Bronchiolitis is an acute viral lower respiratory tract infection. It predominantly affects children younger than two years of age, most commonly during their first year of life [8]. Children may present with a variety of symptoms including but not limited to: persistent spluttery cough, tachypnoea and respiratory distress, low-grade fever and poor feeding. Children younger than six weeks of age may present with episodes of apnoea or cyanosis. On examination, the patient might have evidence of wheezing or crepitations. Alternative diagnosis should be considered if the child has persistent high-grade fever and focal crepitations [5]. 

Risk factors should be highlighted since they play an important part in deciding whether the child requires inpatient care. These include chronic lung disease, haemodynamically significant congenital heart disease, younger than three months, prematurity, history of neuromuscular disorders or immunodeficiency and concerns regarding social situation [5]. 

Patient demographics

Overall, there was an increase in the number of admissions in 2024. This may be attributed to a resurgence of viruses such as RSV following the COVID-19 pandemic, potentially caused by a reduced immune stimulation resulting from lower exposure to circulating pathogens during that period [9-11].

There is a statistically significant difference in gender distribution (p-value 0.008) between the two years, with a higher number of male patients in 2024.

Symptoms

There is a statistically significant difference in the number of patients with coryzal symptoms (p-value 0.0214), spluttery cough (p-value <0.001) and crackles/wheeze (p-value 0.0085) on examination between the two years. Although this is not the case for tachypnoea, there is a trend towards a statistically significant difference. This suggests that in 2024, more patients had a typical picture of bronchiolitis.

Investigations

Despite several studies showing that there is limited benefit of chest X-rays in the diagnosis of acute bronchiolitis, there was a statistically significant difference (p <0.001) between pre-COVID-19 pandemic and post-COVID-19 pandemic and more chest X-rays were performed in the latter [5,9,12]. This results in increased exposure to unnecessary radiation, increase in cost and increase in antibiotic prescription [13]. The use of respiratory viral PCR screen can provide information for epidemiology studies however it does not impact the management of bronchiolitis.

Management

With regards to management of bronchiolitis, several guidelines [5-7] suggest that it is mainly supportive and there is limited evidence to support the use of any medications [5-7]. However, different medications were used in the majority of cases. A high percentage (52.2%, n=47 in 2019, 63.2%, n=67 in 2024) of patients were treated with a short-acting β2 agonist like salbutamol [14]. 

Infants have immature β2 agonist receptor sites; therefore, it is unlikely to be effective and its use is not recommended. The increase in the use of salbutamol shows deviation from NICE, AAP and PREDICT guidelines [5-7]. Haskell et al. and De Brasi et al. have suggested from their studies that factors that may contribute to this include increased turnover of medical staff, reduced senior coverage after hours, time pressure in emergency departments, lack of ‘paediatric confident’ staff and the pressure ‘to do something’ felt by medical doctors [15,16]. While these findings are notable, our audit was not designed to explore causal factors and therefore no conclusions regarding causality can be drawn.

The use of antibiotics should be reserved for patients with clinical signs of secondary bacterial infection or respiratory failure [5-7,16]. Despite this, there was a statistically significant (p-value <0.001) increase in patients with bronchiolitis who were prescribed antibiotics. This may be due to concern of secondary bacterial infections post-COVID. This was also seen in Pop et al.’s retrospective, descriptive, observational study [9]. 

The majority of infants (76.7%; n=69 in 2019, 61.3%; n=65 in 2024) did not require fluid replacement; however, of those who did, only four patients (5%; n=1 in 2019, 7.5%; n=3 in 2024) had replacement through an NGT, which shows deviation from international guideline suggestions. Enteric hydrations should be commenced at two-thirds maintenance and intravenous fluids (IV) should be reserved for those patients who do not tolerate NG hydration. It would be interesting to assess this finding however further studies would need to be carried out to assess whether there are any factors that have contributed to this. There potentially may be reluctance from parents to insert a nasogastric tube for feeds when intravenous fluids may be used as an alternative. In their systematic review, Gill et al. found that there were no differences in the length of stay between enteral tube and IV fluid therapy. However, local complications such as soreness and bruising at insertion site were higher with the IV fluid group [17]. 

Since oxygen therapy is prescribed in severe cases of bronchiolitis, it is possible that the severity of bronchiolitis cases were worse following the pandemic as reflected by the higher amount of patients requiring oxygen therapy post-COVID [11,18]. Pop et al. found similar findings in their retrospective, observational, descriptive study [9]. 

Oxygen therapy might be the only treatment that a child with bronchiolitis needs. Ideally, designated oxygen prescription charts are provided and oxygen therapy is prescribed. According to NICE and PREDICT guidelines, hypoxaemia (SpO2 < 92% for infants younger than six weeks or SpO2 < 90% for infants aged older than six weeks) is an indication to start oxygen [5,7]. An oxygen prescription should include delivery device, flow rate range and target saturations. From the results, it is apparent that there is awareness amongst paediatric trainees for this as there was a statistically significant increase in the amount of treatment plans including oxygen (p-value 0.0010) and treatment charts having oxygen prescribed (0.0084). Nonetheless, 69.5 % (n=73) of patients who were oxygen dependent in 2024 did not have it prescribed on their treatment chart highlighting a clear need for improvement.

This audit shows low adherence with international guidelines. Local guidelines should be introduced with an aim to improve bronchiolitis management and decrease use of unnecessary investigations and medications.

## Conclusions

This audit highlights reveal a significant difference in bronchiolitis management between 2019 and 2024. While documentation and adherence to admission criteria have improved, multiple areas of potential improvement have been highlighted all throughout. The considerable overuse of investigations, as well as treatment that is not supported by evidence are the two key factors. The results suggest that clinical practice has drifted away from international guideline recommendations, with increased use of chest X-rays, salbutamol and antibiotics. A key message is that bronchiolitis remains a clinical diagnosis and most children require only supportive care. Investigations should be kept to a minimum and unnecessary medications avoided. Ultimately, this audit reinforces the importance of introducing local guidelines in Malta. Standardised protocols would support Maltese doctors, reduce variation in practice and ensure that children receive safe, evidence-based care without unnecessary interventions.
